# Deformation Behavior of Al/Cu Clad Composite During Twist Channel Angular Pressing

**DOI:** 10.3390/ma13184047

**Published:** 2020-09-11

**Authors:** Radim Kocich

**Affiliations:** Faculty of Materials Science and Technology, VŠB–Technical University of Ostrava, 70800 Ostrava, Czech Republic; radim.kocich@vsb.cz; Tel.: +420-596-99-44-55

**Keywords:** clad composite, rotary swaging, finite element analysis, effective strain, residual stress

## Abstract

The research and development of modern metallic materials goes hand in hand with increasing their lifetime via optimized deformation processing. The presented work deals with preparation of an Al/Cu clad composite with implemented reinforcing Cu wires by the method of twist channel angular pressing (TCAP). Single and double pass extrusion of the clad composite was simulated numerically and carried out experimentally. This work is unique as no such study has been presented so far. Detailed monitoring of the deformation behavior during both the passes was enabled by superimposed grids and sensors. Both the sets of results revealed that already the single pass imparted significant effective strain (higher than e.g., conventional equal channel angular pressing (ECAP)), especially to the Al matrix, and resulted in notable deformation strengthening of both the Al and Cu composite components, which was confirmed by the increased punch load and decreased plastic flow velocity (second pass compared to first pass). Processing via the second pass also resulted in homogenization of the imposed strain and residual stress across the composite cross-section. However, the investigated parameters featured slight variations in dependence on the monitored location across the cross-section.

## 1. Introduction

As regards to intensive plastic deformation processing, the main focus has been on conventional Fe-based materials, although non-ferrous metals are given attention, too, primarily due to their wide applicability in various industrial branches. However, based on the increasing demands of the industry, the everlasting research of the forming processes increases their applicability also for innovative and newly developed materials. Nevertheless, the aims of the processing are similar regardless of the used material: the materials subjected to intensive plastic deformation are prepared to meet high demands, including the one on having ultra-fine-grained structures (UFG).

Among the preferred methods used to prepare UFG structures within (non)ferrous metals and alloys are severe plastic deformation (SPD) methods, such as equal channel angular pressing (ECAP) [1,2], twist channel (multi) angular pressing (TC(M)AP) [3,4], accumulative roll bonding (ARB) [5], high pressure torsion (HPT) [6], various combinations of torsion and extrusion [7,8], etc. The materials prepared via such methods typically exhibit very high strength at room temperature, but only limited plasticity. The decrease in plastic properties in very fine-grained materials is related to the combination of low deformation strengthening rate, and low value of strain rate sensitivity coefficient (*m*). In other words, the high deformation strengthening rate results in accumulation of dislocations inside the grains, which, in combination with high strain rate sensitivity coefficient *m*, significantly suppresses the development of material failure during deformation processing and thus increases plasticity of the material during forming. Nevertheless, ways how to achieve combinations of high strength and high plasticity exist; such material behavior is characterized as the strength and ductility paradox [9]. The methods leading to the achievement of the paradox are: increasing the imposed strain within UFG materials up to very high values, and performing very short annealing immediately after the intensive deformation processing. Short-time annealing contributes to ordering of defects within grain boundaries, which approaches them to the equilibrium state [9,10]. This phenomenon is crucial to prevent substantial grain growth. In addition, annealing at elevated temperatures imparts decreasing dislocation density (i.e., dislocations annihilation and rearrangement), which facilitates effective development and arrangement of new dislocations, contributing to further deformation strengthening and plasticity increase. Among the first materials, the applicability of the paradox for which was proven (i.e., which exhibited both high strength and superplastic behavior), was the Al4Cu0.5Zr alloy processed via SPD [11].

One of the alternative approaches for achieving favorable combinations of mechanic, electric, or magnetic properties is also preparation of composite materials, among the individual types of which are clad composites [12,13]. Various methods of preparation of the composites, as well as numerous bi-metallic and multi-metallic systems of clad composites, have been reported [14,15,16,17]. However, the majority of the available works focuses on the Cu/Al system. This particular combination of metals exhibits high thermal and electric conductivity (primarily provided by Cu), low density (primarily provided by Al), and the advantage of lower price when compared to single Cu [18,19,20,21,22]. The early researched methods of their preparation were based on bonding under elevated temperatures, e.g., explosive welding [23]. However, such technologies feature heat development during processing and are disadvantageous from the viewpoint of the possible introduction of local structure changes and formation of intermetallic phases negatively affecting not only the mechanic, but also utility properties of the produced clad composite. Moreover, the applicability of such production methods is limited since, if applied for thin sheaths and small billets, the heat-affected region could comprise the entire product.

The methods based on deformation processing, which have so far been applied to prepare clad composites, are e.g., rolling and related methods such as accumulative roll bonding [24] and asymmetrical rolling [25], drawing [26], forward extrusion [27], rotary swaging [28], HPT [29], ECAP [30], and their combinations [31]. The performed experiments revealed that the application of SPD methods leads to a favorable increase in the final utility properties of the produced clad composites. Among the examples is e.g., the Al-Cu system; an Al-Cu composite sheet was reported to be up to 60% lighter and 30–40% cheaper than an identical sheet fabricated from Cu while maintaining comparable thermal and electric conductivity [32]. Moreover, from the viewpoint of mechanical properties, an Al-Cu clad composite can exhibit far higher fracture toughness than the individual metals [33]. Nevertheless, only limited attention has been given to preparation of clad composites via ECAP and ECAP-based methods.

The presented report comprises a detailed study characterizing the deformation behavior of an Al-Cu clad composite during the TCAP process, which enables to impose higher values of effective strain during each pass when compared to conventional ECAP (previous studies dealing with processing of single-phase CP Al billets documented the effectivity of a single pass TCAP to be higher than even double pass ECAP [3,34]). The study features finite element numerical analysis supplemented with results acquired from real experiments consisting of single and double pass extrusion of the designed clad composite (Al sheath with reinforcing Cu wires) via experimental TCAP die (refer to Section 2 for more detailed information). The main focus is on the development of deformation parameters of both the component metals during repeated plastic deformation, as well as on the development of their mechanical properties. Besides, the study is supplemented with characterization of material plastic flow directly influencing the localization and distribution of the effective imposed strain and residual stress.

## 2. Materials and Methods

**Figure 1:**
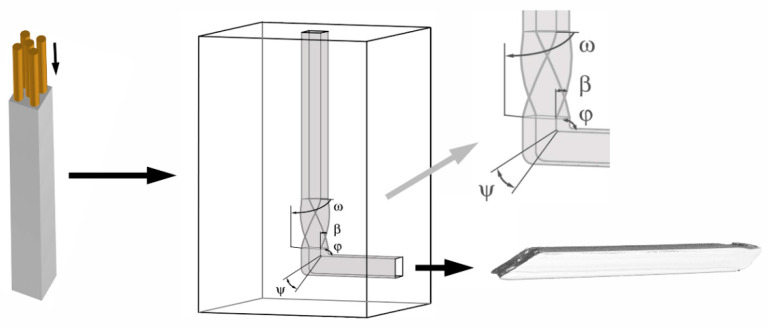
Schematic depiction of manufacturing Al/Cu composite by TCAP.

The aim of the work was to provide a detailed characterization of the effects of TCAP (twist channel angular pressing) processing on the Al/Cu clad composite (Figure 1).

Since the work is unique, as this method has not been used before to process any composite material, the study does not only involve the experimental work, but also numerical analysis. The composite billet consisted of Al sheath and reinforcing Cu wires (Figure 1), and was processed via two subsequent TCAP passes with the selected deformation route *Bc*. The deformation route was based on the *Bc* deformation route known from conventional ECAP, i.e., the composite billet was subjected to +90° rotation before the second pass [35,36]. However, due to the implemented twist, the effects of the deformation route on the billet, moreover a composite one, are expected to be different than those of conventional ECAP. Among the selected investigated structure characteristics and development of mechanical properties, we also investigated selected processing parameters, such as the necessary punch load, values and distributions of the imposed strain, and residual stress.

The first part of the study deals with numerical analysis of extrusion of the clad composite via both the subsequent TCAP passes; the deformation behavior of the composite during processing was studied using Forge NxT commercial software. The simulations were assembled with a model and the geometrical dimensions and mechanical properties of the dies and billet in which were identical to the experimental parameters, which enabled subsequent direct comparison of the predicted results with the experimental ones. The extrusion was carried out on a hydraulic press with the use of MoS_2_ as the lubricant, at the room temperature of approx. 25 °C and extrusion rate of 5 mm s^−1^. The friction was in the simulation determined by the Coulomb friction with the values of µ = 0.02. The values of the mentioned boundary conditions were selected based on the results previously acquired within the study mutual comparison of predicted and experimentally acquired values in which was successfully performed [35]. The geometry of the die used in the simulation was defined by the following angles: *ω* = 90° (twist rotation angle), *φ* = 90° (channel bending angle), *β* = 40° (twist slope angle), ***ψ*** = 20° (angle of the arc of curvature in which the two channels intersect) (Figure 1).

The second part of the study deals with practical realization of the multiple TCAP process. The selected materials were commercially pure Cu (99.97%) with the chemical composition of 0.0074 Ni, 0.0058Sn, 0.0030Zn, 0.0031Fe, 0.0023Si, bal. Cu (in wt.%), and commercially pure Al (99.97%) with the chemical composition of 0.125Fe, 0.10Si, 0.020Cu, 0.020Zn, 0.015Mg, 0.015Mn, 0.015Ti, bal. Al (in wt.%). Before completing the composite, both the component metals were pre-annealed in a furnace at 500 °C for 30 min. The dimensions of the samples prepared for extrusion were matching the parameters given in the simulation, i.e., square 12 mm × 12 mm cross-section and 130 mm length.

The structure analyses of the extruded clad composite samples were performed via the electron backscatter diffraction (EBSD) method (scanning electron microscopy (SEM)). Preparations of the samples for the analyses were carried out by grinding on SiC papers and final electrolytic polishing. SEM investigations were done using a Tescan Lyra 3 FIB/SEM microscope equipped with a NordlysNano EBSD camera (Oxford Instruments, Abingdon-on-Thames, Great Britain). The EBSD scans were acquired on samples tilted by 70° with the steps of 50 nm and the accelerating voltage of 20 kV. To enable comparison with the predicted results, the presence of residual stress in the structures of the extruded billets was evaluated by analyzing the internal grains misorientations in the rainbow color scheme in the scale from 0° (negligible presence of residual stress), to 15° (occurrence of residual stress). Overall structure scan of the cross-section of the final extruded billet was performed on the polished sample using the OLYMPUS DSX1000 digital microscope (Shinjuku, Tokyo, Japan).

Last but not least, the mechanical properties of the composites were investigated via microhardness measurement performed on transversal cross-sectional cuts using a Zwick/Roell testing machine (Zwick/Roell, Ulm, Germany). The applied load was 200 g, and the loading time per indent was 10 s.

### Numerical Simulation

**Figure 2:**
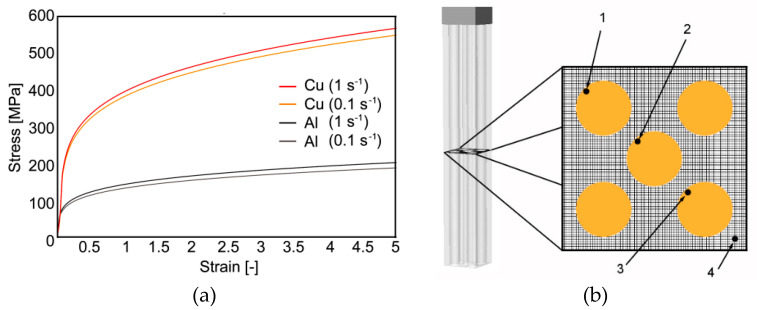
Stress-strain curves used for FEM (finite element method) computations (**a**); placement of analyzed sections within extruded composite (**b**).

The deformation behavior of both the component metals was predicted with the use of an elastic-plastic model defined via the Newton–Raphson convergent algorithm. The mesh of the clad composite billet consisted of 215,871 nodes. The billet was meshed with tetrahedral elements, whereas both the extruder and die were considered as rigid parts. Since severe shear deformations were expected to occur during the simulation, automatic re-meshing was activated. The stress-strain curves, depicted in Figure 2a, acquired for the experimentally used materials were determined on the basis of torsion tests performed using SETARAM, a servo-hydraulic torsion plastometer, at room temperature with the strain rates of 0.1 and 1 s^−1^.

The experimentally acquired stress-strain data were entered into the material flow stress database of the computational software. The Haensel–Spittel equation (Equation (1)) was then used to characterize material behavior during deformation processing:

(1) $$\sigma_{f}=Ae^{m_{1}T}T^{m_{8}}\varepsilon^{m_{2}}e^{m_{4}/\varepsilon }{\left(1+\varepsilon \right)}^{m_{5}T}e^{m_{6}\varepsilon }{\dot{\varepsilon }}^{m_{3}}{\dot{\varepsilon }}^{m_{7}T}$$

where *ε* is the equivalent von Mises strain, *T* is the temperature, $\dot{\varepsilon }$ is the equivalent von Mises strain rate, and *A*, *m*_1_, *m*_2_, *m*_3_, *m*_4_, *m*_5_, *m*_6_, *m*_7_, *m*_8_, and *m*_9_ are regression coefficients. The values of the individual coefficients for Cu are, respectively, 411.19 MPa, −0.00121, 0.21554, 0.01472, −0.00935, and *m*_5_ ÷ *m*_8_ are 0. The values of the individual coefficients for Al are, respectively, 151.323 MPa, −0.00253, 0.21142, 0.03177, −0.00654, *m*_5_÷*m*_8_ are 0.

The boundary conditions defined in the simulation were the temperature of 25 °C, and the values of parameters describing the temperature behavior of aluminum and copper, i.e., Young’s modulus, Poisson’s ratio, thermal expansion, thermal conductivity, heat transfer coefficient, specific heat, emissivity, and density. The parameters were defined for Al as the constants of 72 (GPa), 0.3, 2.4 × 10^−5^ (K^−1^), 250 (W/(mK)), 1230 (J/kgK) 0.03 and 2800 (kg/m^3^), and for Cu as 111 (GPa), 0.3, 1.7 × 10^−5^ (K^−1^), 394 (W/(m K)), 100,000 (W/m^2^ K), 398 (J/kg K), 0.7, and 8960 (kg/m^3^).

In order to provide more specific characterization of the material plastic flow of both the composite components, a monitoring grid was superimposed through the billet being extruded, perpendicularly to the longitudinal axis of the composite (Figure 2b). The deformation behavior of the composite was monitored via four individual monitoring sensors localized within the superimposed grid (sensors 1–4, see Figure 2b). To enable detailed evaluation of the monitored phenomena, the superimposed grid was designed with very small square cells (0.1 × 0.1 mm). The purpose of this grid was to provide the possibility to characterize the influence of different plastic flows of the individual component metals on the development of temperature and its magnitude during processing, the development of the effective imposed strain and its (in)homogeneity across the billet cross-section, as well as the values and localization of residual stress. This research method is effective for characterization of the influences of the individual deformation zones within the die on the processed material.

## 3. Results

### 3.1. Temperature Development and Imposed Strain

**Figure 3:**
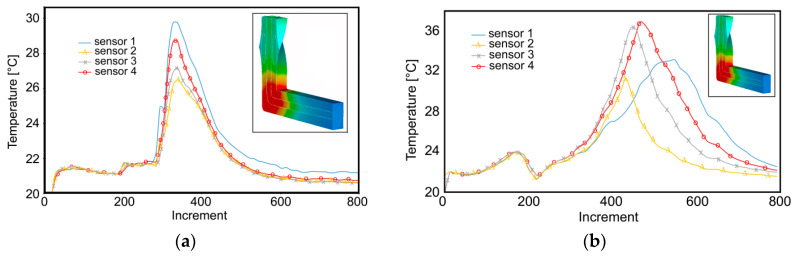
Development of temperature during: first pass (**a**); second pass (**b**).

As documented by the predicted results, the component metals exhibited differences in their behaviors during plastic deformation. The imposed strain is an important factor, the distribution of which non-negligibly influences also the distribution of temperature. However, for the Al/Cu clad composite, the temperature development was affected not only by the particular component metal, but also by the localization of the individual monitoring sensor in the deformation zones (Figure 3).

The maximum value of temperature was detected in the main deformation zone (MDZ) for both the passes (see the detail in Figure 3). As documented by the temperature-time dependences for the monitored sensors, the maximum temperature value was approx. 50% higher than the average processing temperature during the first pass (Figure 3a). However, during the second pass, the maximum temperature increased up to ~37 °C (Figure 3b). The increase in the maximum temperature during the second pass can primarily be attributed to the effect of plastic deformation, i.e., deformation strengthening, of the Cu wires featuring higher flow stress than the Al sheath [37].

The occurring migration of the maximum temperature value is an intriguing phenomenon; whereas sensor 1 exhibited the maximum temperature during the first pass, sensor 4 exhibited the maximum temperature during the second pass (Figure 3a,b). This phenomenon was related to the applied deformation route *Bc*. In other words, the maximum temperatures occurred in the upper half of the cross-section of the extruded billet for both the passes, since the quicker plastic flow occurring in this region imparts the most intense shear. While sensors 1 and 2 were localized within the upper half of the cross-section during the first pass, during the second pass, as the result of the 180° rotation, sensor 4 was localized in the upper half of the billet cross-section.

**Figure 4:**
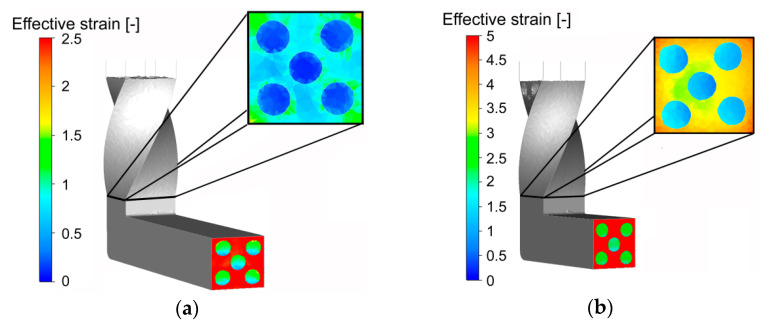
Effective imposed strain during: first pass (**a**); second pass (**b**).

By the effect of the inserted Cu wires, the effective strain was high also along the cross-sectional diagonals, although the wires themselves were not substantially affected by the imposed strain. The development of distribution of the imposed strain was rather complicated, since it was influenced by multiple factors. During the first pass, the twist deformation zone (TDZ) of the die affected primarily the corner areas of the extruded composite (Figure 4a) (except at mutual Al/Cu interfaces).

The effect of the imposed strain on the Cu wires started to be evident after passing through the MDZ. As can be seen in Figure 4a, contrary to the Cu wires, the Al sheath exhibited more or less homogeneous distribution of high values (~2.5) of the effective strain after the first pass. The significantly lower effective strain values occurring within the Cu wires were caused by the minor influence of the TDZ on the wires; the strain gradient occurring between the Al sheath and Cu wires could not be completely eliminated during subsequent passing through the MDZ. On the other hand, the significant increase in the values of the imposed strain within the Cu wires occurring after passing through the MDZ points to the non-negligible deformation effect of this zone on both the composite components. The favorable combination of both the deformation zones within the die thus finally contributed to the more or less homogeneous distribution of the effective imposed strain throughout the composite cross-section after the second pass.

The imposed strain development was different during the second TCAP pass, the deformation effect of the TDZ during which was more notable than during the first pass. The predicted differences in the values of the imposed strain across the Al sheath cross-section were significantly lower, i.e., homogenization of the distribution of the effective strain occurred (Figure 4b). After passing through the MDZ, the Al sheath evidently exhibited increase in the values of the homogeneously distributed imposed strain (~5), whereas the Cu wires exhibited notable homogenization of the effective strain distribution (rather than significant increase in the imposed strain values).

**Figure 5:**
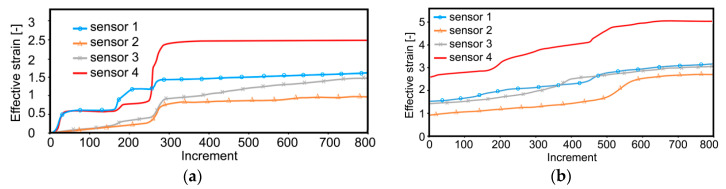
Imposed strain in individual sensors during: first pass (**a**); second pass (**b**).

As demonstrated by the predicted developments of the effective strain values in the individual monitored sensors within the composite, the developments of the imposed strain exhibited differences related to the individual component metal (similar to temperature behavior, see Figure 3a,b). During the first TCAP pass, the maximum values of the imposed strain were detected in sensor 4, i.e., in the Al sheath (Figure 5a).

The significant differences in the values of the imposed strain between the Al sheath and Cu wires originated primarily in the variations in the plastic flow intensity of both the component metals (Section 3.2).

Whereas during the first pass, the most significant effect on the imposed strain could be observed for the TDZ (jump increase in the imposed strain values were evident especially in sensors 1 and 4, see Figure 5a), during the second pass, the imposed strain in the monitored sensors exhibited gradual increasing (Figure 5b). This phenomenon was most probably related to the deformation strengthening of both the metals occurring during the first pass, by the effect of which the Al sheath became more effective transmitter of the imposed strain, which gradually diminished the differences in the values of the imposed strain within the individual wires. The predicted results showed that the second pass resulted in significant increase and homogenization in the imposed strain values, based on previous deformation strengthening. The Al sheath, being more susceptible to the imposed strain and thus exhibiting more intense plastic flow, featured higher effective strain values than the Cu wires. On the other hand, the differences in the values of the imposed strain between the individual Cu wires resulted from their localization. The most intense shear within the Cu was detected in sensor 1, i.e., in the upper half of the extruded composite.

The effective strain in sensors 2 and 3 exhibited relatively rapid increases in their developments during the second pass (Figure 5b). Whereas for sensor 3, this increase originated from its relocation caused by the selected deformation route, for sensor 2, this increase originated most probably from the occurring strengthening of the Al sheath in its vicinity. For the first pass, the moment in which the monitored sensors passed through the MDZ could clearly be identified (Figure 5a—increment 250 featured notable imposed strain jump increase). Despite the fact that minor jump increases in the imposed strain could be detected also during the second pass, their values were lower than during the first pass. On the contrary, most of the monitored locations (especially the Cu wires) exhibited gradual increases in the imposed strain.

### 3.2. Plastic Flow

**Figure 6:**
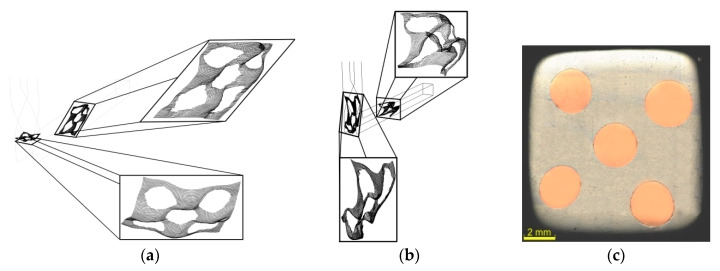
Material plastic flow of Al/Cu composite during: first pass (**a**), second pass (**b**); optical microscopy image of cross-sectional cut through billet extruded via two passes (**c**).

Monitoring of the plastic flow behavior enabled detailed characterization of the influences of both the deformation zones on the extruded composite; their effects on the effective imposed strain were not identical, however, both the sections influenced non-negligibly not only the values of the imposed strain, but also its distribution. Already during the first TCAP pass, the TDZ evidently affected not only the (sub)surface, but also the axial region of the extruded composite (Figure 6a).

The effects of the TDZ were evident also on the Al/Cu interfaces, the superimposed grids at which exhibited evident serrations originating from rotations of the clad composite. Moreover, the original monitoring plane exhibited deformation in the extrusion direction. In other words, the deformations of the individual cells of the superimposed grid were not only caused by the tangential plastic flow, but also by its axial component which became dominant after passing through the MDZ (Figure 6a). The prediction thus revealed that significant plastic flow occurred within virtually all the regions of the Al sheath already during the first TCAP pass. On the other hand, the material flow in the upper half of the Al sheath was dominant. Together, with the significantly slower plastic flow of the axial Cu wire (decelerating effect), this phenomenon resulted in more intense slipping of both the metals in this region, and consequently in inhomogeneous distribution of the imposed strain, especially across the cross-sections of the Cu wires (as also confirmed by Figure 4a and Figure 5a).

The cells of the superimposed grid, heavily deformed during the first pass, were subsequently deformed even more during the second pass. The effect of the previous deformation strengthening contributed to relatively uniform deformation of the entire superimposed grid; this was observed also for the Cu wires, the plastic flow of which was supported by the substantial plastic deformation introduced to the Al sheath. The second pass also resulted in full bonding of both the components, as can be seen in the optical microscopy scan of a cross-sectional cut through the extruded billet in Figure 6c; the metals did not exhibit slipping during processing.

**Figure 7:**
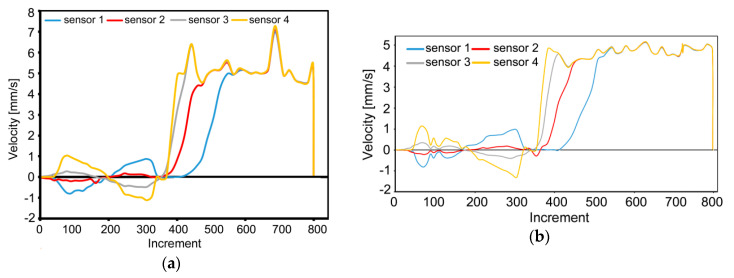
Development of plastic flow velocity during: first pass (**a**); second pass (**b**).

The TDZ introduced rotations also during the second pass. However, during this pass, the rotations affected more intensively the axial region of the composite. Overall rotation of the cross-section of the billet, by 180°, was introduced by the selected deformation route *Bc*, which contributed to substantial reduction of the gradient of the imposed strain across the Cu wires cross-sections. This was also confirmed by the plastic behavior of the superimposed grid, the typical skewness of the cells of which reduced after passing through the MDZ (when compared to the first pass), as well as by the overall velocities of the plastic flow in the individual monitored sensors depicted in Figure 7a,b for the first and second TCAP pass, respectively.

The graphical dependencies in Figure 7a,b depict the velocities of movement of the individual sensors in the monitored plane in relation to the extrusion direction (i.e., axis of the horizontal channel). The sensors enabled monitoring of the values and vectors of plastic flow velocities in the individual locations of the composite during the entire extrusion. As can be seen, passing of the composite through the TDZ caused reversion of the vector of plastic flow velocity for all the sensors during both, the first and second pass (Figure 7a,b). In other words, the negative values of velocity demonstrate the period of extrusion of the monitored volume of the material (i.e., the monitored sensor) in which reversed against the horizontal extrusion direction. Intriguing was also the notable decrease in the overall plastic flow velocities of the composite occurring between the first and second TCAP pass. This fact can be attributed to the deformation strengthening introduced during the first TCAP pass, which resulted not only in the decrease in the values of plastic flow velocity for all the sensors, but also in reduction of the observed oscillations.

### 3.3. Residual Stress

**Figure 8:**
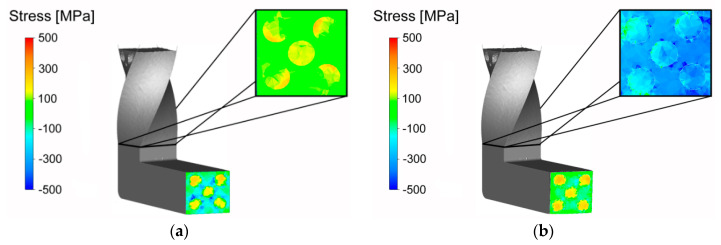
Distribution of residual stress during: first pass (**a**); second pass (**b**).

The effects of both the TCAP passes on the composite components were different from the viewpoint of residual stress. The composite exhibited prevailing tensile stress during the first pass though the TDZ. This phenomenon can be attributed to two main factors, the first one of which was the rotational movement of the Al sheath, the intensity of which decreased towards the axial region of the billet. The second factor was the dominant plastic flow occurring in the axial region of the composite (see Figure 6a). As the result of these factors, the maximum values of tensile stress were detected in the peripheral regions of the Cu wires (Figure 8a) after the first pass through the TDZ.

The stress state within the composite changed after passing through the channel bending, i.e., the MDZ. However, the stress character changed gradually from dominant tensile stress to dominant compressive stress during passing through the channel zone between the TDZ and MDZ due to the increasing resistance against the plastic flow caused by gradual filling of the MDZ (and the horizontal channel part) with the composite material. Behind the MDZ, the stress character changed again as axial differences in the plastic flows in different composite regions started to be notable. As evident from Figure 8a, the Cu wires in the extruded billet predominantly exhibited tensile stress, whereas the Al sheath in the extruded billet primarily featured compressive stress.

The development of residual stress was different during the second pass. The Cu wires featured local presence of tensile stress before entering the MDZ also during the second pass. On the other hand, the Al sheath exhibited predominant occurrence of compressive stress from the very beginning of extrusion. With continuing filling of the MDZ, the tensile stress within the Cu wires gradually transformed to compressive stress (Figure 8b); the gradual changing of the stress character was observed between the individual deformation zones, similarly to the first pass. After passing through the MDZ, homogenization of stress within both the Al sheath and Cu wires occurred. Nevertheless, the stress development was opposite for both the materials. Whereas the Cu wires featured gradual increase and, at the same time, homogenization of tensile stress, the Al sheath featured gradual homogenization of compressive stress. Contrary to the first TCAP pass, the occurring homogenization of stress decreased the overall stress gradient for both the component metals. Among the primary causes of this behavior was the occurrence of vortex-like flow within the Al sheath during extrusion [35]. In other words, the supplementary twist zone (compared to conventional ECAP) contributed to homogenization of the stress distribution throughout the composite cross-section. Indisputable influence had also the selected deformation route.

**Figure 9:**
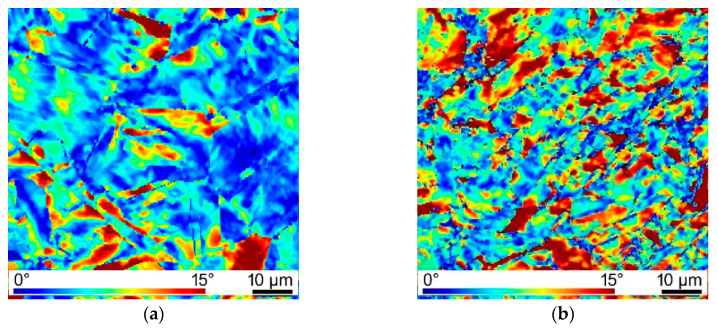
Maps of internal grains misorientations indicating residual stress within peripheral Cu wire of composite billet extruded via: first pass (**a**); second pass (**b**).

Experimental verification of the predicted data was performed via analyses of internal grains misorientations within both the components of the billet extruded via two consequent TCAP passes. The results of the analyses for the peripheral Cu wire of the billets extruded via single and double TCAP pass are depicted in Figure 9a,b, respectively.

As can be seen, both the figures feature more or less homogeneous distribution of the areas featuring high misorientations (i.e., red color), pointing to the presence of residual stress. However, the occurrence of residual stress was more notable in the structure of the Cu wire from the composite extruded via two TCAP passes, which is in accordance to the predicted results (Figure 8a,b). In addition, the structure of the wire extruded via two TCAP passes exhibited finer grains and substructure development imparted by the severe imposed shear strain (see also Figure 4b).

### 3.4. Punch Load and Microhardness

The maximum loading force during the first pass reached to 40 kN (Figure 9). Whereas passing of the billet through the first deformation zone (TDZ) only resulted in a slight increase in the punch load, passing of the billet through the MDZ led to a significant increase in this parameter; the rapid increase in the punch load occurring approx. after 7 s of extrusion was followed with a mild linear increase to the maximum punch load value achieved in the time of extrusion of approx. 15 s. Such a steep increase in the loading force was most probably related to the increasing flow stress, primarily of the Cu wires. The subsequent decrease in the punch load after 15 s of extrusion was caused by gradual emptying of the twist zone of the channel and consequent decrease in friction between the Al sheath and channel walls.

The development of the punch load during the second TCAP pass featured even steeper increasing character from the very beginning of extrusion. Such behavior was imparted by two main influencing factors, both of which affected the development of the punch load during the entire pass. The first factor was the accumulation of deformation strengthening within both the component metals during the previous pass. In addition, the strain imposed during the first TCAP pass resulted in the changes of shapes of the ends of the inserted Cu wires (not shown here), which was the second influencing factor. In the moment in which the billet entered the twist deformation zone, the previous deformation strengthening accumulated in the Al sheath, but primarily in the Cu wires, resulted in the increase in the punch load by up to 50% (when compared to the first pass).

The already mentioned deformation strengthening, contributing also to the notable increase in the punch load during the second pass, was experimentally observed via microhardness measurements carried out along the diagonals across the cross-sectional cuts through both the composite billets (Figure 9b). The initial HV values of the original annealed component metals were 37.4 for Al, and 58.6 for Cu. The microhardness values evidently increased after both, the single and double TCAP pass. However, the absolute increase in the HV values was more significant after the first pass. After the second pass, the increase in the HV values was less notable, however, the Cu wires within the billet extruded via route *Bc* exhibited the highest (maximum observed) HV values.

## 4. Discussion

The study featuring processing of the Al/Cu clad composite via the TCAP method revealed variations when compared to processing of single-component billets. Firstly, the deformation behavior of the investigated composite was influenced by the combination of the characteristic properties of both the component metals. For example, the comparison of the presented results with the results acquired during studies dealing with processing of single Al [34], or Cu [35], shows different temperature developments. Combining the Al and Cu within a single billet resulted in inhomogeneous temperature distribution, primarily due to the higher flow stress and thermo-physical parameters of the Cu wires (compared to the Al sheath). Moreover, the increase in temperature introduced by the intensive plastic flow of the Al sheath (especially in locations the vortex-like flow in which occurs [38,39,40]), generating heat originated from friction with the die and with the individual wires, should also be taken into account.

The deformation behavior, especially the effective imposed strain and plastic flow, of the composite was affected not only by the individual component metals, but also by the deformation zones within the die and the selected deformation route. Although the effects of the individual deformation zones on the values of the effective imposed strain are not equal, their mutual effect, which makes TCAP an advantageous method for processing of multicomponent materials, is non-negligible. Passing of the composite through the TDZ did not introduce as high strain as passing through the MDZ. The MDZ primarily influenced the axial region of the extruded billet, whereas in the TDZ, the highest effective strain values were detected in the (sub)surface regions of the Al sheath. The inter-regions and the axial region of the billet were more or less affected by the imposed strain in the TDZ, too, but the values of the imposed strain were significantly lower within the Cu wires. However, as the result of the variations in the velocities of the axial plastic flow of both the materials in the TDZ, the overall gradients in the observed parameters between the (sub)surface and axial billet regions were more or less reduced. In other words, among the main contributions of the TDZ is that it diminishes the differences between the axial and (sub)surface regions of the extruded composite. Therefore, the TDZ primarily contributes to homogenization of the imposed strain across the cross-section of the clad composite (especially the Cu wires).

During TCAP, the locations of the individual Cu wires with respect to the die channel vary, which is not possible for conventional ECAP [41]. By this reason, the plastic flow of the individual Cu wires is aggravated/supported during passing through and behind the MDZ. This behavior is closely related to the observed differences in the effective strain and residual stress values and distributions. Certain inhomogeneity of the imposed strain across the Cu wires cross-sections was also related to their orientations in the individual deformation zones during both the TCAP passes. Nevertheless, the final observed homogeneity of the imposed strain and residual stress was affected positively also by the applied deformation route (rotation of the billet with respect to the individual deformation zones resulted in alternation of the Cu wires and suppression of the differences occurring in the deformation history), as well as by the occurring deformation strengthening, which also affected the mentioned increase in temperature observed during the second pass.

**Figure 10:**
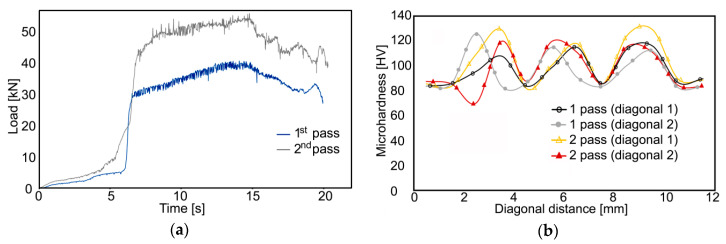
Material plastic flow of Al/Cu composite during both passes (**a**); microhardness measured experimentally across cross-sections of extruded billets (**b**).

The time dependence of the punch load featured notable oscillations for both the passes, however, the oscillations were more intense during the second pass (see Figure 10a).

Such serrated development is typically related to plastic flow instability [34,35], which was, in the presented study, most probably the direct effect of the different deformation strengthening (plastic flow) of the component metals. This supposition is supported by the fact that the oscillations were the most notable when the billet was passing through the MDZ, during which mutual slipping of both the component metals initiated by the differences in their plastic flows was the most intense. As discussed, the plastic flow of the Al sheath was more intense and the flows of the Cu wires exhibited localized tendencies to “delay”; this difference was caused by the Cu having higher flow stress than Al. The presupposition of deformation strengthening of both the component metals was confirmed by the data acquired from microhardness testing.

## 5. Conclusions

The presented study documented the results of numerical and experimental analysis of successful room-temperature preparation of an Al/Cu clad composite by single and double pass of twist channel angular pressing (TCAP). Both the TCAP passes introduced significant shear strain to the component metals, as the maximum effective imposed strain reached up to the value of 5 for the Al sheath of the billet extruded by two passes. The imposed strain contributed to deformation strengthening resulting not only in the increase in punch load by almost 50% and increase in microhardness up to 130 HV for the Cu wires, but also in temperature increase during processing. Nevertheless, the processing temperature was still only ~37 °C during the second pass, i.e., the processing conditions were safe from the viewpoint of introduction of possible structure changes. The results also showed that the processing via multiple passes increased the bonding quality of both the metals, as both the distributions of the imposed strain and residual stress homogenized after the second pass, which was ensured by the favorable combination of twist and bending deformation zones within the single unique die.

## References

[1] Kocich R., Kursa M., Szurman I., Dlouhý A. (2011). The influence of imposed strain on the development of microstructure and transformation characteristics of Ni–Ti shape memory alloys. J. Alloys Compd..

[2] Kunčická L., Kocich R., Drápala J., Andreyachshenko V.A. FEM simulations and comparison of the ecap and ECAP-PBP influence on Ti6Al4V alloy’s deformation behavior. Proceedings of the Metal 2013—22nd International Conference on Metallurgy and Materials.

[3] Kocich R., Kunčická L., Král P., Macháčková A. (2016). Sub-structure and mechanical properties of twist channel angular pressed aluminium. Mater. Charact..

[4] Kocich R., Kunčická L., Macháčková A. (2014). Twist Channel Multi-Angular Pressing (TCMAP) as a method for increasing the efficiency of SPD. IOP Conf. Ser. Mater. Sci. Eng..

[5] Jamaati R., Naseri M., Toroghinejad M.R. (2014). Wear behavior of nanostructured Al/Al2O3 composite fabricated via accumulative roll bonding (ARB) process. Mater. Des..

[6] Kawasaki M., Foissey J., Langdon T. (2013). Development of hardness homogeneity and superplastic behavior in an aluminum–copper eutectic alloy processed by high-pressure torsion. Mater. Sci. Eng. A.

[7] Kocich R., Szurman I., Kursa M., Fiala J. (2009). Investigation of influence of preparation and heat treatment on deformation behaviour of the alloy NiTi after ECAE. Mater. Sci. Eng. A.

[8] Khosravifard A., Jahedi M., Yaghtin A.H. (2012). Three dimensional finite element study on torsion extrusion processing of 1050 aluminum alloy. Trans. Nonferrous Met. Soc. China.

[9] Valiev R.Z., Alexandrov I.V., Zhu Y.T., Lowe T.C. (2002). Paradox of Strength and Ductility in Metals Processed Bysevere Plastic Deformation. J. Mater. Res..

[10] Humphreys F., Hatherly M. (2004). Recrystallization and Related Annealing Phenomena.

[11] Langdon T. (2006). Grain boundary sliding revisited: Developments in sliding over four decades. J. Mater. Sci..

[12] Cetin A., Krebs J., Durussel A., Rossoll A., Inoue J., Koseki T., Nambu S., Mortensen A. (2011). Laminated Metal Composites by Infiltration. Met. Mater. Trans. A.

[13] Polyanskii S.N., Kolnogorov V.S. (2002). Cladded Steel for the Oil and Gas Industries. Chem. Pet. Eng..

[14] Motarjemi A.K., Kocak M., Ventzke V. (2002). Mechanical and fracture characterization of a bi-material steel plate. Int. J. Press. Vessel. Pip..

[15] Jin J.Y., Hong S.I. (2014). Effect of heat treatment on tensile deformation characteristics and properties of Al3003/STS439 clad composite. Mater. Sci. Eng. A.

[16] Movahedi M., Kokabi A., Reihani S.S. (2011). Investigation on the bond strength of Al-1100/St-12 roll bonded sheets, optimization and characterization. Mater. Des..

[17] Inoue J., Sadeghi A., Kyokuta N., Ohmori T., Koseki T. (2017). Multilayer Mg: Stainless Steel Sheets, Microstructure, and Mechanical Properties. Metall. Mater. Trans. A.

[18] Kocich R., Kunčická L., Davis C.F., Lowe T.C., Szurman I., Macháčková A. (2016). Deformation behavior of multilayered Al–Cu clad composite during cold-swaging. Mater. Des..

[19] Kunčická L., Kocich R., Dvořák K., Macháčková A. (2019). Rotary swaged laminated Cu-Al composites: Effect of structure on residual stress and mechanical and electric properties. Mater. Sci. Eng. A.

[20] Kim I.K., Hong S.I. (2013). Effect of heat treatment on the bending behavior of tri-layered Cu/Al/Cu composite plates. Mater. Des..

[21] Lee T.H., Lee Y.J., Park K.T., Jeong H.G., Lee J.H. (2015). Mechanical and asymmetrical thermal properties of Al/Cu composite fabricated by repeated hydrostatic extrusion process. Met. Mater. Int..

[22] Li F.S., Xu R.Z., Wei Z.C., Sun X.F., Wang P.F., Li X.F., Li Z. (2020). Investigation on the Electron Beam Welding of Al/Cu Composite Plates. Trans. Indian Inst. Met..

[23] Fronczek D., Wojewoda-Budka J., Chulist R., Sypien A., Korneva A., Szulc Z., Schell N., Zieba P. (2016). Structural properties of Ti/Al clads manufactured by explosive welding and annealing. Mater. Des..

[24] Kim I.K., Hong S.I. (2013). Roll-Bonded Tri-Layered Mg/Al/Stainless Steel Clad Composites and their Deformation and Fracture Behavior. Metall. Mater. Trans. A.

[25] Li X., Zu G., Ding M., Mu Y., Wang P. (2011). Interfacial microstructure and mechanical properties of Cu/Al clad sheet fabricated by asymmetrical roll bonding and annealing. Mater. Sci. Eng. A.

[26] Jee M.H., Choi J.U., Park S.H., Jeong Y.G., Baik D.H. (2012). Influences of tensile drawing on structures, mechanical, and electrical properties of wet-spun multi-walled carbon nanotube composite fiber. Macromol. Res..

[27] Kazanowski P., E Epler M., Misiolek W.Z. (2004). Bi-metal rod extrusion—Process and product optimization. Mater. Sci. Eng. A.

[28] Kocich R., Kunčická L., Macháčková A., Šofer M. (2017). Improvement of mechanical and electrical properties of rotary swaged Al-Cu clad composites. Mater. Des..

[29] Ma X., Huang C., Xu W., Zhou H., Wu X.L., Zhu Y. (2015). Strain hardening and ductility in a coarse-grain/nanostructure laminate material. Scr. Mater..

[30] Jafarlou D., Zalnezhad E., Ezazi M., Mardi N., Hassan M. (2015). The application of equal channel angular pressing to join dissimilar metals, aluminium alloy and steel, using an Ag–Cu–Sn interlayer. Mater. Des..

[31] Sapanathan T., Khoddam S., Zahiri S.H., Zarei-Hanzaki A. (2014). Strength changes and bonded interface investigations in a spiral extruded aluminum/copper composite. Mater. Des..

[32] Kwon H.C., Jung T.K., Lim S.C., Kim M.S., Kwon H.C. (2004). Fabrication of Copper Clad Aluminum Wire (CCAW) by Indirect Extrusion and Drawing. Mater. Sci. Forum.

[33] Kim H., Hong S.I. (2015). Deformation and fracture of diffusion-bonded Cu–Ni–Zn/Cu–Cr layered composite. Mater. Des..

[34] Kunčická L., Kocich R., Král P., Pohludka M., Marek M. (2016). Effect of strain path on severely deformed aluminium. Mater. Lett..

[35] Kocich R., Fiala J., Szurman I., Macháčková A., Mihola M. (2011). Twist-channel angular pressing: Effect of the strain path on grain refinement and mechanical properties of copper. J. Mater. Sci..

[36] Macháčková A. (2020). Decade of Twist Channel Angular Pressing: A Review. Mater. Basel.

[37] Russell A., Lee K.L. (2005). Structure-Property Relations in Nonferrous Metals.

[38] Orlov D., Beygelzimer Y., Synkov S., Varyukhin V., Tsuji N., Horita Z. (2009). Microstructure Evolution in Pure Al Processed with Twist Extrusion. Mater. Trans..

[39] Kocich R., Greger M., Kursa M., Szurman I., Macháčková A. (2010). Twist channel angular pressing (TCAP) as a method for increasing the efficiency of SPD. Mater. Sci. Eng. A.

[40] Kocich R., Kunčická L., Král P., Strunz P. (2018). Characterization of innovative rotary swaged Cu-Al clad composite wire conductors. Mater. Des..

[41] Djavanroodi F., Ebrahimi M. (2010). Effect of die channel angle, friction and back pressure in the equal channel angular pressing using 3D finite element simulation. Mater. Sci. Eng. A.

